# Radiologic manifestations of angioedema

**DOI:** 10.1007/s13244-014-0329-1

**Published:** 2014-05-03

**Authors:** Kousei Ishigami, Sarah L. Averill, Janet H. Pollard, Joshua M. McDonald, Yutaka Sato

**Affiliations:** Department of Radiology, University of Iowa Hospitals and Clinics, 3885 JPP, 200 Hawkins Drive, Iowa City, IA 52242 USA

**Keywords:** Angioedema, Upper airway obstruction, Glossomegaly, Head and neck angioedema, Visceral angioedema

## Abstract

**Objectives:**

The purpose of this pictorial review is to present imaging findings of angioedema involving the various organs.

**Conclusion:**

The role of imaging for patients with angioedema includes the evaluation of the upper airway for obstruction and the exclusion of other possible aetiologies, such as neoplastic or infectious processes. Glossomegaly is a common finding of head and neck angioedema. Angioedema may involve organ systems beyond the superficial regions and the head and neck including the gastrointestinal and genitourinary tracts. Angioedema of the visceral organs is often accompanied by adjacent fluid, and it is commonly diffuse or concentric but can also be multifocal and asymmetric.

***Teaching Points*:**

- *The evaluation of the upper airway obstruction is important for head and neck angioedema.*
- *Glossomegaly with decreased attenuation is common in head and neck angioedema.*
- *Angioedema of the visceral organs can be multifocal and asymmetric.*
- *Angioedema of the visceral organs is often accompanied by adjacent fluid.*
- *It is important to include clinical and laboratory findings for the diagnosis of angioedema.*

## Introduction

Angioedema was first described by J.L. Milton in 1876. First named angioneurotic oedema by Quinckein 1882, it is sometimes referred to as Quincke’s oedema [1, 2]. There are various causes of angioedema [3]. The incidence of angioedema varies with its causes. For example, the estimated incidence of angiotensin-converting enzyme (ACE) inhibitor-induced angioedema is 0.1 to 1.0 % [4] and that of hereditary angioedema (HAE) ranges from 0.002 to 0.01 % [5]. It is clinically characterised as episodic localised and transient oedematous swelling, most commonly affecting superficial regions such as the face, genitals, and extremities. If angioedema only affected the superficial regions, imaging evaluation would not be necessary. Angioedema, however, may affect any part of the body, and intra-abdominal involvement can even occur without cutaneous involvement [3, 6, 7]. Angioedema can also present with life-threatening respiratory distress when the upper airway is involved [8]. Angioedema involving the gastrointestinal tract may present as an acute abdominal pain resulting in unnecessary laparotomy [2, 9]. Although the diagnosis of angioedema is clinical, awareness of this entity and its characteristic imaging findings by radiologists will aid in raising the suspicion for angioedema, which may warrant further clinical workup and prevent unnecessary surgery. Additionally, for patients with known previous history of angioedema who have abdominal pain, contrast-enhanced CT of the abdomen and pelvis is the study of choice to evaluate for possible visceral involvement as well as to rule out other pathology.

The purpose of this pictorial review is to present imaging findings of angioedema involving the various organs. Differential diagnoses and complications of angioedema are discussed.

### Classification and pathogenesis of angioedema

Classification of angioedema is summarised in Table 1.

**Table 1:** Classification of angioedema

I. Idiopathic
II. Medications (allergic or non-allergic) ACE inhibitors: activated bradykinin ↑ NSAIDs: COX 1 inhibition → leukotrienes ↑
III. Allergen-induced (foods, etc.): histamine ↑ (IgE mediated)
IV. Physically induced (cold, pressure, vibration, ultraviolet, etc.): histamine ↑ (direct)
V. Deficiency or inactivation of C1-INH : uncontrolled complement activation → bradykinin ↑ 1. HAE a. HAE type 1 (80–85 %): decreased level of C1-INH b. HAE type 2 (10–15 %): normal or high levels of dysfunctional C1-NIH 2. AAE with C1-INH deficiency a. AAE type 1 associated mainly with lymphoproliferative disorders (e.g., lymphoma) b. AAE type 2 associated with anti-C1-INH autoantibodies (e.g., autoimmune diseases)
VI. HAE type 3 (rare): normal or slightly low C1-INH level with normal function 1. Coagulation factor XII gene mutation (HAE-FXII): activated factor XII ↑ → bradykinin ↑ 2. HAE-unknown

*ACE* angiotensin-converting enzyme, *NSAIDs* nonsteroidal anti-inflammatory drugs, *COX1* cyclooxygenase 1, *C1-INH* complement 1 esterase inhibitor, *HAE* hereditary angioedema, *AAE* acquired angioedema

The majority of cases of angioedema are idiopathic. When a trigger is identified, most often it is a medication, allergen, or a physical agent such as pressure or cold [3]. Many medications can cause angioedema but those most commonly implicated are angiotensin-converting enzyme (ACE) inhibitors and nonsteroidal anti-inflammatory drugs (NSAIDs) [3].

A less common, but important cause of angioedema is deficiency or inactivation of C1 esterase inhibitor (C1-INH) in the complement system, which can be either hereditary [5] or acquired [10]. Hereditary angioedema (HAE) is autosomal dominant, and is classified into three types [5, 10]. Acquired angioedema (AAE), a very rare condition is associated mainly with lymphoproliferative disorders and autoimmune diseases [10].

The mechanism of angioedema is the overproduction or failure to inactivate vasoactive stimulants such as histamine, bradykinin, and leukotrienes (Table 1), which lead to increased vascular permeability. Allergen-induced angioedema is due to histamine mediated by immunoglobulin E (IgE) [2, 3]. ACE inhibitor-induced angioedema is not entirely understood, but it is postulated that the use of ACE inhibitors increases bradykinin levels [2, 3], which is a potent vasodilator. Given the estimated incidence of ACE inhibitor-induced angioedema of 0.1 to 1.0 % [4] and the large number of patients on ACE inhibitors, one theory is that affected individuals have an underlying partial deficiency of C-1 esterase [2]. NSAIDs inhibit cyclooxygenase 1 (Cox 1), which results in overproduction of leukotrienes [2]. HAE types 1 and 2 and AAE (deficiency or inactivation of C1-INH) are characterised by uncontrolled complement activation and resultant increased bradykinin production [2, 3, 5, 10]. In addition, a rare form of HAE (type 3) mainly noted in female patients is associated with a mutation in the coagulation factor XII gene [11], which also ultimately results in increased levels of bradykinin.

Angioedema attacks can last from 1 to 5 days depending on the causes and symptoms [2]. Importantly, acute attacks of HAE and AAE do not respond to epinephrine, antihistamines, or steroid [2]. Consequently, treatment is often mainly supportive, consisting of intravenous fluids and upper airway management. Allergen and drug induced angioedema are treated with elimination of causative agents [2, 12]. For acute attacks of HAE and AAE, C1-INH concentrate, kallikrein inhibitor ecallantide, bradykinin B2 antagonist icatibant, or fresh frozen plasma are administered [2, 3, 9, 10].

### Imaging findings

#### Head and neck

**Fig. 1:**
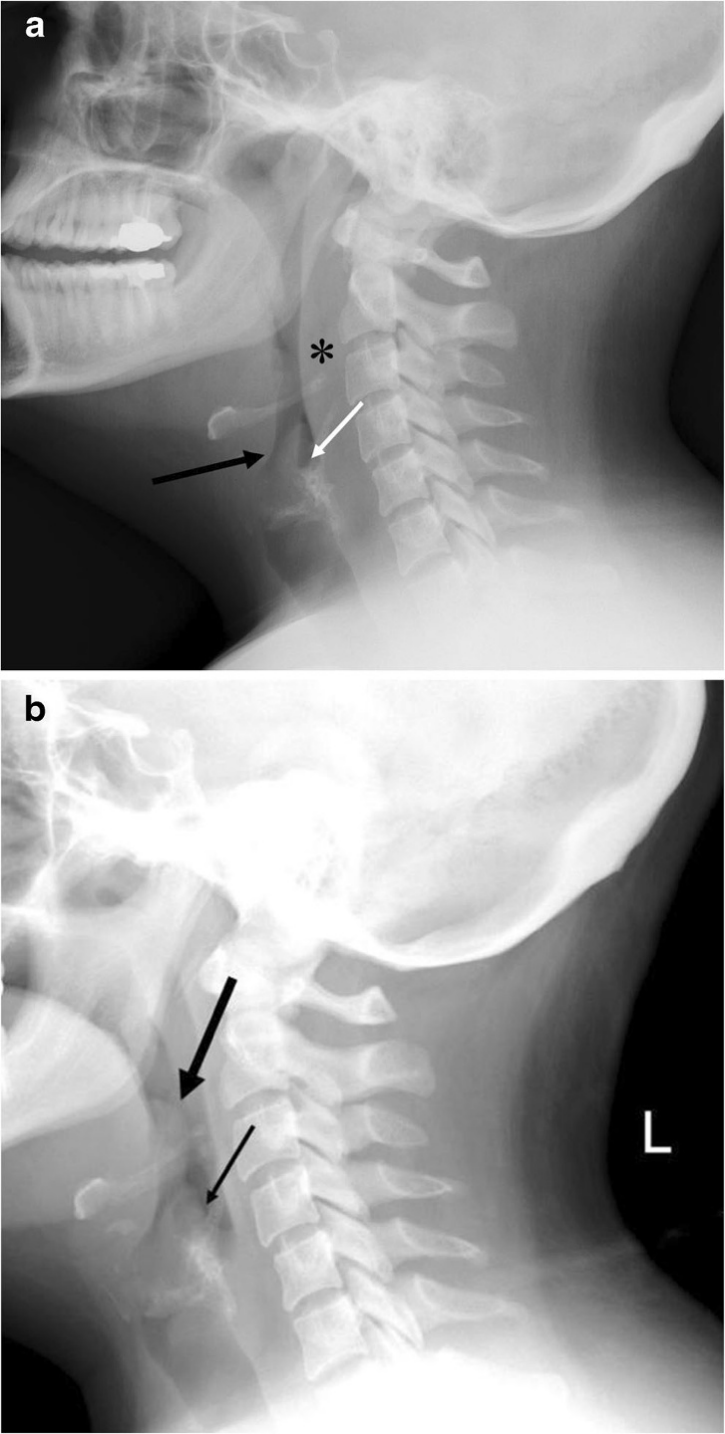
A 33-year-old female with hereditary angioedema (HAE). Soft tissue swelling of the neck during recurrent angioedema attacks. **a** The patient complained of neck swelling. Lateral radiograph of the neck shows thickening of the retropharyngeal soft tissue (*asterisk*), enlargement of the epiglottis with narrowing of the vallecula (*large arrow*), and thickening of the aryepiglottic folds (*small arrow*). **b** Six months later, the patient again complained of neck swelling and swallowing difficulty. Lateral view of the neck plain film shows enlargement of the epiglottis (*large arrow*, thumb sign) and thickening of the aryepiglottic fold (*small arrow*). No retropharyngeal soft tissue thickening is noted

**Fig. 2:**
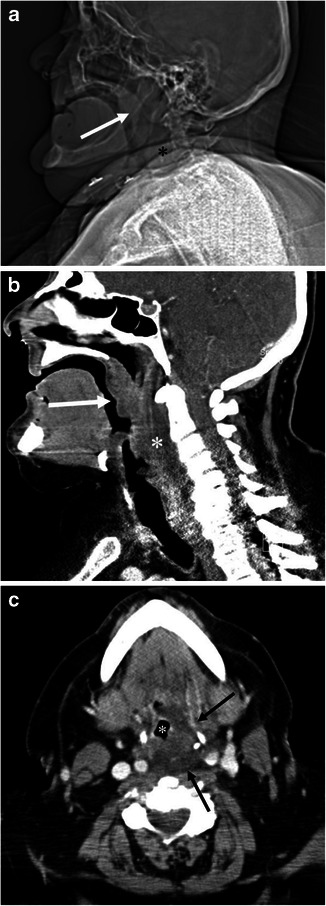
A 68-year-old male with angiotensin converting enzyme (ACE) inhibitor-induced angioedema involving the soft palate, pharynx and larynx. **a** Scout view of the neck computed tomography (CT) shows enlargement of the soft palate (*arrow*) and thickening of the retropharyngeal soft tissue (*asterisk*). **b** The sagittal reformatted contrast enhanced (CE)-CT shows marked swelling of the soft palate (*arrow*) and posterior pharyngeal wall (*asterisk*) with decreased attenuation. **c** The axial CE-CT image shows swelling of the left posterolateral retropharyngeal space (*arrows*), and narrow displaced pharyngeal airway (*asterisk*)

Upper airway obstruction can be a life-threatening complication from angioedema. In emergent cases, airway management precludes imaging. In less urgent cases, lateral plain radiograph of the neck could be used for the initial imaging assessment (Fig. 1). On plain film, care should be paid to the presence or absence of swelling of the retropharyngeal soft tissue, epiglottis, aryepiglottic folds, and soft palate [13]. Computed tomography (CT)—with lateral scanogram of the neck and multiplanar reformatted (MPR) images—is also useful in the evaluation upper airway obstruction (Fig. 2).

**Fig. 3:**
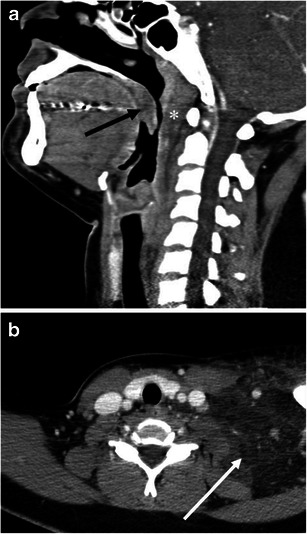
A 24-year-old female with systemic lupus erythaematosus and anti-phospholipid syndrome presenting with acquired angioedema. **a** The sagittal reformatted CE-CT shows diffuse retropharyngeal (*asterisk*) and soft palate oedema (*arrow*) causing upper airway encroachment at the level of the oropharynx. **b** The axial CE-CT shows extensive soft tissue oedema with fat stranding of the left lower neck (*arrow*)

**Fig. 4:**
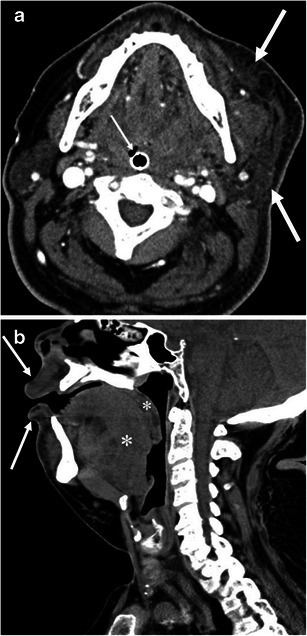
A 64-year-old male with ACE inhibitor-induced angioedema with airway obstruction. **a** The axial CE-CT image shows left hemifacial swelling with subcutaneous fat stranding (*large arrows*). The patient is status post placement of an endotracheal tube (*small arrow*). **b** One year later, the patient developed recurrent angioedema after tooth extraction. The sagittal reformatted image of the unenhanced CT shows marked swelling of the tongue base and soft palate (*asterisks*). Also noted is swelling of the lips (*arrows*)

**Fig. 5:**
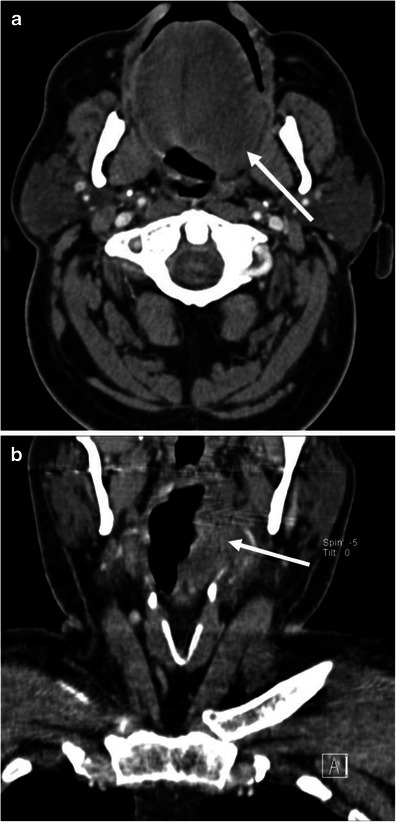
A 62-year-old male with ACE inhibitor-induced angioedema. **a** The axial image of CE-CT demonstrates asymmetric left-sided tongue oedema (*arrow*). **b** The coronal reformatted image of CE-CT shows extension of oedema into the left pharyngeal wall (*arrow*)

**Fig. 6:**
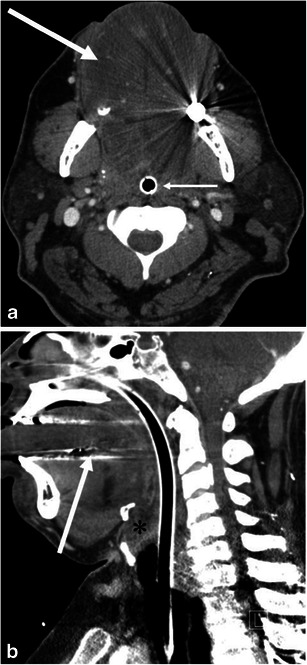
A 49-year-old male with ACE inhibitor-induced angioedema. **a** The axial CE-CT shows diffuse enlargement of the tongue with decreased attenuation due to oedema (*large arrow*). *Small arrow* indicates the endotracheal tube. **b** Sagittal CE-CT shows glossomegaly (*arrow*) and marked laryngeal oedema (*asterisk*). The endotracheal tube is inserted by the trans-nasal approach because of marked glossomegaly

**Fig. 7:**
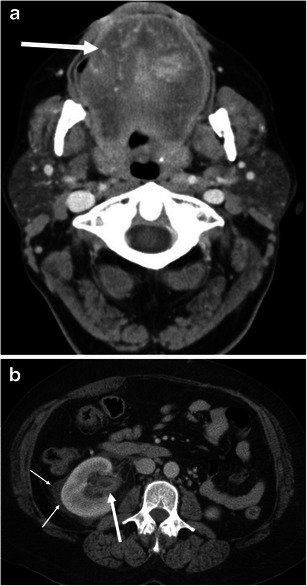
A 54-year-old female with ACE inhibitor-induced angioedema. **a** The axial CE-CT of the neck shows diffuse enlargement of the tongue (*arrow*). Vessels are recognised within the tongue, reflecting decreased attenuation due to oedema. **b** The axial CE-CT of the abdomen on the same date shows thickened and contrast-enhancing uroepithelium of the renal pelvis (*large arrow*). Perinephric fluid is also noted (*small arrows*). No stone disease was recognised in the ureter or urinary bladder (not shown). Urinalysis was unremarkable except for microscopic haematuria, which returned to normal 3 days later. Follow-up CT 1 month later showed complete resolution of renal pelvic wall thickening and perinephric fluid (not shown)

On CT, facial or neck swelling with subcutaneous fat stranding is occasionally recognised (Figs. 3 and 4). The swelling is usually diffuse but can be focal or asymmetric. Similarly, narrowing of the upper airway and tongue swelling can be focal or asymmetric (Figs. 2 and 5). Glossomegaly (swelling of the tongue) is a common finding of angioedema (Figs. 6 and 7), with isolated glossomegaly reportedly more common in patients with ACE inhibitor-induced angioedema than HAE [5].

**Fig. 8:**
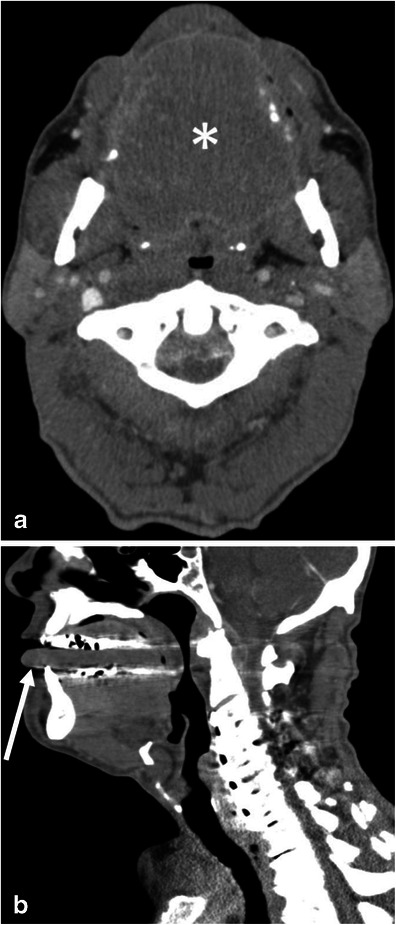
A 64-year-old male with amyloidosis presenting with diffuse tongue swelling. **a** The axial CE-CT shows the tongue to be diffusely swollen (*asterisk*). Note the attenuation of the tongue is soft tissue density. Glossomegaly caused by angioedema typically shows low density (see Figs. 6 and 7). **b** The sagittal CE-CT shows diffuse tongue swelling with anterior protrusion of the tip of the tongue (*arrow*)

The differential diagnosis of upper airway obstruction includes neoplasms, parapharyngeal abscess, and infection (e.g., tonsillitis, laryngitis, and cellulitis) and these should be excluded by the imaging findings, laboratory data, clinical course, and laryngoscopy [14, 15]. Glossomegaly may also be seen in patients with amyloidosis (Fig. 8). Angioedema-induced glossomegaly typically shows a low-density tongue, while the one caused by amyloidosis shows soft tissue density. In addition, angioedema has an acute onset with short duration whereas amyloidosis is chronic.

Although it is rare, cases of central nervous system involvement have been reported in patients with HAE and ACE inhibitor-induced angioedema [16–18]. Imaging findings vary from unremarkable to extensive white matter oedema [16–18].

#### Body

**Fig. 9:**
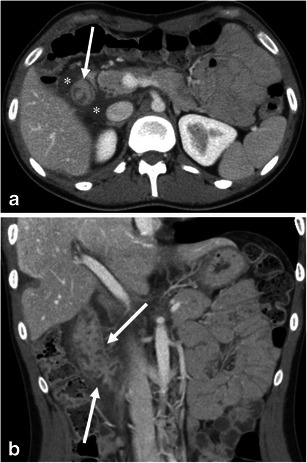
A 26-year-old female with HAE involving the 2nd portion of the duodenum. **a** The axial CE-CT shows the duodenal wall to be thickened (*arrow*) with surrounding free fluid (*asterisks*). **b** The coronal reformatted image shows oedematous thickening of the duodenal folds (*arrows*)

**Fig. 10:**
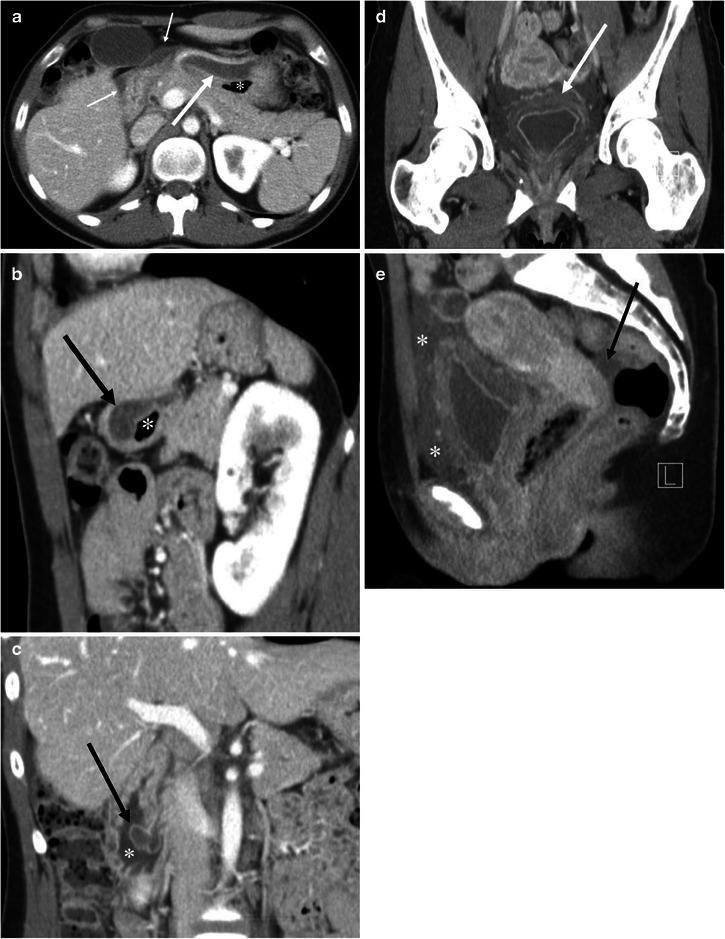
A 27-year-old female with HAE involving the stomach, ampulla of Vater, urinary bladder, and small and large bowel (not shown). **a** The axial CE-CT shows marked submucosal oedema of the anterior wall of the stomach (*arrow*). *Asterisk* indicates the lumen of the stomach. Fluid is noted adjacent to the stomach and duodenum (*small arrows*). **b** The sagittal reformatted image clearly shows asymmetrical gastric wall involvement of angioedema (*arrow*). *Asterisk* indicates the lumen of the stomach. **c** Coronal reformatted image shows marked oedematous swelling of the ampulla of Vater (*arrow*). *Asterisk* indicates the duodenal lumen. **d** The coronal reformatted image of the pelvis shows mucosal enhancement and extensive submucosal oedema of the urinary bladder (*arrow*). Urinalysis was unremarkable without evidence of urinary tract infection. **e** The sagittal reformatted image shows fluid around the urinary bladder in the extraperitoneal space (*asterisks*). *Arrow* indicates a small amount of intraperitoneal free fluid in the cul-de-sac

**Fig. 11:**
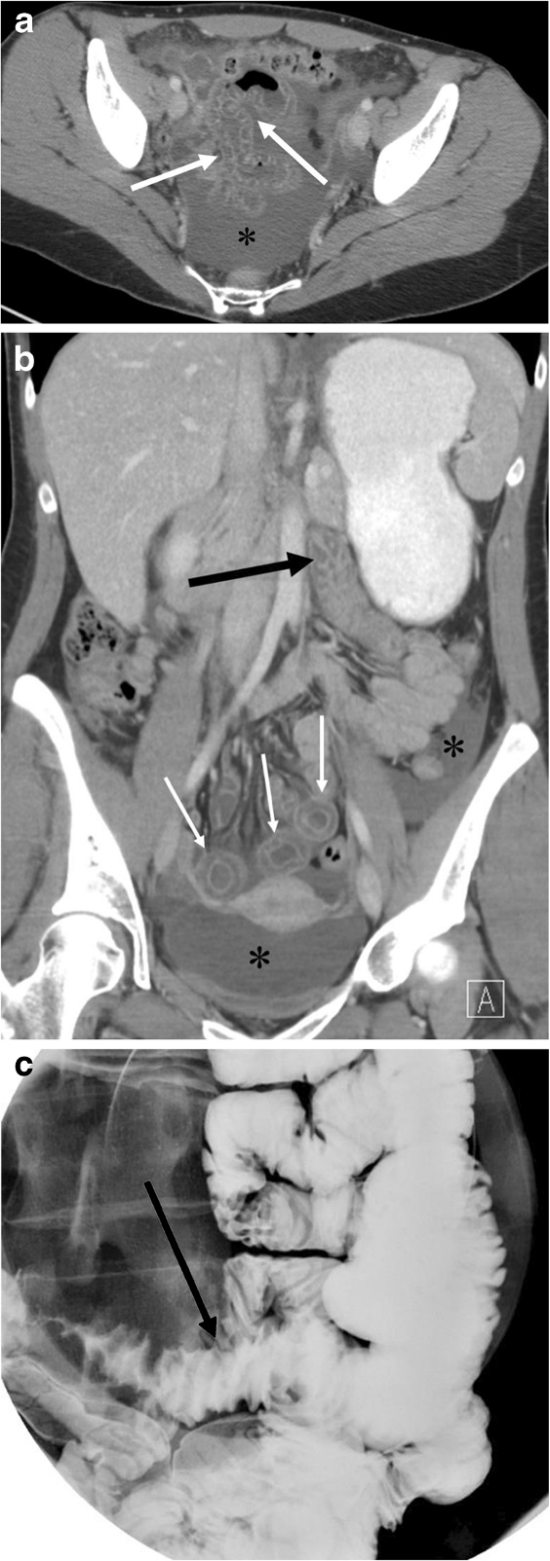
A 29-year-old female with ACE inhibitor-induced angioedema involving multiple small bowel loops. **a** The axial CE-CT shows oedematous thickening of the small bowel folds with mucosal enhancement (*arrows*). A moderate amount of intraperitoneal free fluid is noted in the pelvis (*asterisk*). **b** The coronal reformatted image demonstrates oedematous wall thickening of the proximal jejunum and intrapelvic small bowel. The proximal jejunum (*large arrow*) shows oedematous thickening of the jejunal folds. The short axis section of the involved intrapelvic small bowel shows the halo sign, representing a low-density submucosal layer with mucosal and subserosal enhancement (*small arrows*). *Asterisks* denote intraperitoneal free fluid. **c** Spot film of single-contrast small bowel barium study shows straight small bowel folds thickening (*arrow*) and a stack of coins appearance

**Fig. 12:**
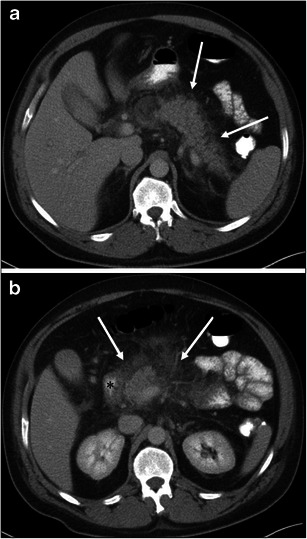
A 44-year-old male with HAE presenting with acute pancreatitis. **a** The axial CE-CT image shows fat stranding around the pancreatic body and tail (*arrows*). **b** At the level of the pancreatic head, there is more extensive peripancreatic fat stranding (*arrows*). Wall thickening of the 2nd portion of the duodenum (*asterisk*) is seen, although it could be caused by pancreatitis

Gastrointestinal involvement can be seen in HAE, AAE, and ACE inhibitor-induced angioedema [2, 7, 9, 19, 20] (Figs. 9, 10, and 11). In addition, a case of small bowel angioedema triggered by intravenous iodinated contrast medium has been reported [21]. Gastrointestinal involvement of angioedema clinically presents as acute abdominal pain, nausea, vomiting, diarrhea, and various degrees of obstruction. A flare of angioedema can even predispose patients to acute pancreatitis likely because of duct obstruction and/or oedema of the ampulla of Vater [22] (Figs. 10 and 12).

Abdominal radiographs are of limited value for the evaluation of abdominal involvement of angioedema. Contrast-enhanced CT is the imaging modality of choice for patients with possible angioedema involving the gastrointestinal tracts. MPR of the involved bowel can show mural stratification or the halo sign because of a thickened low-density submucosal layer reflecting submucosal oedema with mucosal and subserosal enhancement (Fig. 11) [7, 20]. Involved small bowel folds show regular thickening with low density (Figs. 9 and 11). This finding has also been likened to a stack of coins on barium studies because of the uniformly thickened folds with a relatively parallel arrangement [23] (Fig. 11). Oedematous bowel wall thickening is commonly accompanied by adjacent free fluid (Figs. 9 and 10). It should be emphasised that wall thickening may not always be circumferential and may be asymmetric (Fig. 10). In addition, gastrointestinal involvement can be multifocal (Fig. 11). Oral contrast is not necessary in emergent situations and also may obscure mural stratification of the involved bowel wall [7].

Angioedema attacks can last from 1 to 5 days without treatment [2], and intestinal angioedema typically subsides in 12–24 h [3]. Recurrent episodes of unexplained acute abdominal pain (e.g., three or more episodes within 3–6 months [3]) and the imaging finding of oedematous bowel wall thickening may warrant an initial workup for angioedema including detailed family and medication (e.g., ACE inhibitors) histories and laboratory evaluation of the complementary system. Although imaging follow-up may not usually be indicated, complete remission of abnormal imaging findings may support the clinical diagnosis of angioedema, and vice versa.

**Fig. 13:**
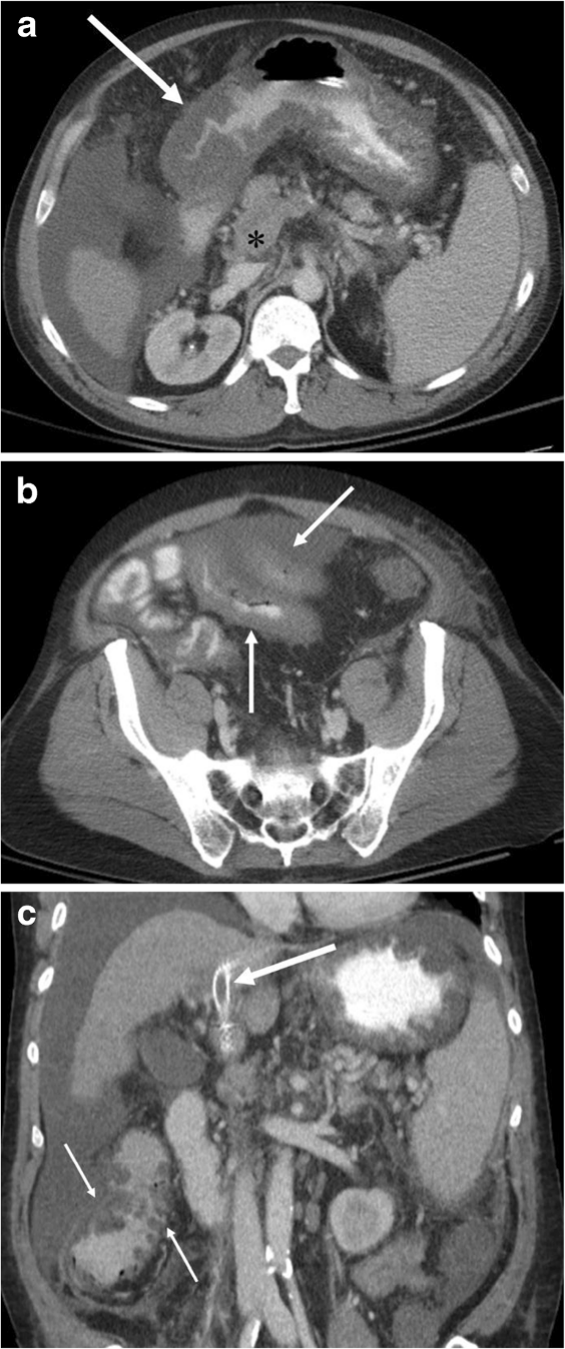
A 55-year-old male with liver cirrhosis presenting with portal venous thrombus and resultant small bowel ischaemia and congestion of the gastrointestinal tracts. **a** The venous phase of the axial CE-CT shows marked oedematous wall thickening of the gastric antrum (*arrow*). The splenoportal confluence (*asterisk*) is poorly opacified. Ascites and splenomegaly are also noted. **b** The axial CE-CT at the level of the pelvis shows wall thickening of the small bowel (*arrows*). Laparotomy found necrosis of the small bowel. **c** The coronal reformatted image shows an occluded transjugular intrahepatic portosystemic shunt (TIPS) stent (*large arrow*). The portal vein is not opacified at the level of the hepatic hilum due to thrombus. The *ascending colon* showed oedematous wall thickening (*small arrows*)

**Fig. 14:**
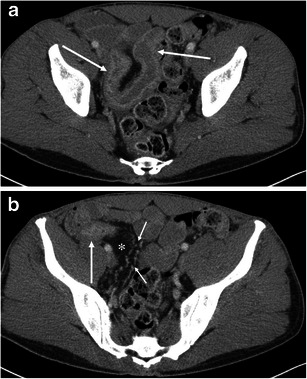
A 42-year-old male with Crohn’s disease. **a** The axial CE-CT shows wall thickening and hyperenhancement of the terminal ileum (*arrows*). The layer of wall enhancement is thick and irregular. **b** The terminal ileum near the ileocecal valve shows wall enhancement and luminal narrowing (*arrow*). Creeping fat (*asterisk*) and engorgement of the vasa recta (*small arrows*) are noted

Differential diagnoses of gastrointestinal angioedema consist of other pathologies that may cause bowel wall oedema, including ischaemia, vasculitis, and hypoproteinaemia [7, 23]. Since there are numerous entities that may present as mural stratification or the halo sign, the diagnostic approach is based on the exclusion of other potential causes clinically and radiologically. For example, it is possible to suggest venous ischaemia based on the presence of mesenteric venous thrombus (Fig. 13). However, making the differential diagnosis of intestinal angioedema versus non-occlusive mesenteric ischaemia may be difficult based solely on imaging findings because both entities show a halo sign without mesenteric fat stranding [24]. In non-occlusive mesenteric ischaemia, diffuse narrowing of mesenteric arterial branches may be recognised [25], and patients with non-occlusive mesenteric ischaemia present clinically with hypovolaemia, hypotension, and low cardiac output [24]. Associated skin findings may be recognised in patients with vasculitis such as lupus enteritis and Henoch-Schönlein purpura [7, 26]. In Crohn’s enteritis, mesenteric fat stranding, engorgement of vasa recta (comb sign), luminal narrowing, and the degree of wall enhancement are more striking than intestinal angioedema (Fig. 14). Additionally, the presence of fistula, abscess, and findings to suggest coexisting chronic inflammation such as submucosal fatty infiltration and creeping fat may be helpful for the differential diagnosis. Furthermore, clinical history and laboratory data can exclude radiation enteritis, hypoproteinaemia, portal hypertensive enteropathy, and infection.

Genitourinary involvement may be rarely encountered. The clinical symptom of renal involvement may simulate renal colic [5]. Urinary bladder involvement clinically presents as lower abdominal pain, urinary stammering, and urinary bladder retention [5]. Similar to gastrointestinal involvement of angioedema, renal and urinary bladder involvement can show oedematous wall thickening with adjacent fluid (Figs. 7 and 10). Although imaging findings may mimic those of urinary tract infection, it is possible to exclude this by clinical symptoms and urinalysis.

Pulmonary involvement of angioedema has not been well documented. A case of acute respiratory distress syndrome has been reported [27]. Chest symptoms may be associated with upper airway obstruction or oesophageal involvement such as breathing or swallowing difficulties [5]. Chest radiographs are utilised for the monitoring of endotracheal tube placement, lung aeration, and the presence or absence of pleural effusion and air space diseases.

## Conclusions

The major role of imaging of angioedema in the head and neck is to evaluate for the presence of upper airway obstruction and exclusion of other causes. Glossomegaly with low attenuation is commonly present in head and neck angioedema. Angioedema may involve the gastrointestinal tracts and more rarely the genitourinary system. Angioedema of the visceral organs is often accompanied by adjacent fluid, and the involvement may be multifocal or asymmetric (may not always be diffuse or concentric). As the clinical presentation can mimic surgical and inflammatory causes of abdominal pain, the exclusion of these aetiologies is an important role in the imaging evaluation for visceral organ involvements in patients who present with angioedema in any organ system.

## References

[1] Quinke HE (1882). Über akutes umschriebenes Hautödem. Monatsh Prakt Dermatol.

[2] de Graaff LC, van Essen M, Schipper EM, Boom H, Duschek EJ (2012). Unnecessary surgery for acute abdomen secondary to angiotensin-converting enzyme inhibitor use. Am J Emerg Med.

[3] Frigas E, Nzeako UC (2002). Angioedema. Pathogenesis, differential diagnosis, and treatment. Clin Rev Allergy Immunol.

[4] Vleeming W, van Amsterdam JG, Stricker BH, de Wildt DJ (1998). ACE inhibitor-induced angioedema. Incidence, prevention and management. Drug Saf.

[5] Bork K, Meng G, Staubach P, Hardt J (2006). Hereditary angioedema: new findings concerning symptoms, affected organs, and course. Am J Med.

[6] Wolpin BM, Weller PF, Katz JT, Levy BD, Loscalzo J (2009). Clinical problem-solving. The writing on the wall. N Engl J Med.

[7] Vallurupalli K, Coakley KJ (2011). MDCT features of angiotensin-converting enzyme inhibitor-induced visceral angioedema. AJR Am J Roentgenol.

[8] Ko CH, Ng J, Kumar S, Hurst M (2006). Life-threatening angioedema in a patient with systemic lupus. Clin Rheumatol.

[9] Jung M, Rice L (2011). “Surgical” abdomen in a patient with chronic lymphocytic leukemia: a case of acquired angioedema. J Gastrointest Surg.

[10] Cicardi M, Zanichelli A (2010). Acquired angioedema. Allergy Asthma Clin Immunol.

[11] Bork K, Wulff K, Hardt J, Witzke G, Staubach P (2009). Hereditary angioedema caused by missense mutations in the factor XII gene: clinical features, trigger factors, and therapy. J Allergy Clin Immunol.

[12] Muller BA (2004). Urticaria and angioedema: a practical approach. Am Fam Physician.

[13] Poon CM, Koenigsberg RA, Betsy A, Izes BA (1997). Angioedema due to angiotensin-converting enzyme inhibitor use: radiographic findings in 3 patients. Emerg Radiol.

[14] Kuo GP, Torok CM, Aygun N, Zinreich SJ (2011). Diagnostic imaging of the upper airway. Proc Am Thorac Soc.

[15] Raman SP, Lehnert BE, Pruthi S (2009). Unusual radiographic appearance of drug-induced pharyngeal angioedema and differential considerations. AJNR Am J Neuroradiol.

[16] Sunder TR, Balsam MJ, Vengrow MI (1982). Neurological manifestations of angioedema. Report of two cases and review of the literature. JAMA.

[17] Decloedt E, Freercks R, Maartens G (2009). Cerebral angioedema associated with enalapril. Br J Clin Pharmacol.

[18] HoxHa M, Meta D, Kalo T (2013). Hereditary angioedema as a potential cause of cerebral edema. Otorhinolaryngologia Head Neck Surg.

[19] Marmery H, Mirvis SE (2006). Angiotensin-converting enzyme inhibitor-induced visceral angioedema. Clin Radiol.

[20] Scheirey CD, Scholz FJ, Shortsleeve MJ, Katz DS (2011). Angiotensin-converting enzyme inhibitor-induced small-bowel angioedema: clinical and imaging findings in 20 patients. AJR Am J Roentgenol.

[21] Chen CK, Chang HT, Chen CW, Lee RC, Sheu MH, Wu MH, Chou HP, Shen YC, Chiu NC, Chang CY (2012). Dynamic computed tomography of angioedema of the small bowel induced by iodinated contrast medium: prompted by coughing-related motion artifact. Clin Imaging.

[22] Matesic D, Fernández Pérez ER, Vlahakis NE, Hagan JB (2006). Acute pancreatitis due to hereditary angioedema. Ann Allergy Asthma Immunol.

[23] Levine MS, Rubesin SE, Laufer I (2008). Pattern approach for diseases of mesenteric small bowel on barium studies. Radiology.

[24] Furukawa A, Kanasaki S, Kono N, Wakamiya M, Tanaka T, Takahashi M, Murata K (2009). CT diagnosis of acute mesenteric ischemia from various causes. AJR Am J Roentgenol.

[25] Ofer A, Abadi S, Nitecki S, Karram T, Kogan I, Leiderman M, Shmulevsky P, Israelit S, Engel A (2009). Multidetector CT angiography in the evaluation of acute mesenteric ischemia. Eur Radiol.

[26] Lee CK, Ahn MS, Lee EY, Shin JH, Cho YS, Ha HK, Yoo B, Moon HB (2002). Acute abdominal pain in systemic lupus erythematosus: focus on lupus enteritis (gastrointestinal vasculitis). Ann Rheum Dis.

[27] da Costa JT, da Silva JM, Cunha L, Castel-Branco MG, Azevedo MV (1994). Hereditary angioedema presenting with adult respiratory distress syndrome. Chest.

